# Correlation between Post-COVID-19, Chemosensitive Function, Blood Group, and Oral Health-Related Quality of Life

**DOI:** 10.1155/2022/8715777

**Published:** 2022-05-11

**Authors:** Rehab Abdulwahab M. Alabsi, N. C. Sandeepa, Rema Tariq Misfer, Majdah Mahmood Alraqdi, Mohammed Ibrahim M. Hamdi

**Affiliations:** ^1^King Khalid University, College of Dentistry, Abha, Saudi Arabia; ^2^Department of Diagnostic Sciences and Oral Biology, King Khalid University, College of Dentistry, Abha, Saudi Arabia

## Abstract

**Materials and Methods:**

A cross-sectional information on demographics, symptomatic disease status, ABO blood group, and oral health-related quality of life (OHRQoL) was collected among 100 patients who were earlier tested positive for COVID-19 reverse transcription-quantitative polymerase chain reaction (RT-qPCR) and were now reporting to the College of Dentistry for routine treatment after recovery. Objective evaluation of olfactory and gustatory disturbances was elicited using the Connecticut Chemosensory Clinical Research Center (CCCRC) test and gustatory function testing. Furthermore, OHRQoL was assessed using Oral Health Impact Profile (OHIP-14).

**Results:**

More than half of the patients (62%) had some form of olfactory dysfunction/alteration, and 42% had poor CCCRC scores. About 14% reported ageusia, while 68% reported some form of taste alterations, and 55% reported poor OHRQoL. A statistically significant difference was reported between different ABO blood groups and subjective loss of smell (*p* < 0.05). The subjective loss of taste, CCCRC score, and dysgeusia were found to be independent of OHIP-14 (*p* > 0.05), but the taste intensity score was dependent on OHIP 14 (*p* < 0.05). Moreover, a majority (70.8% and 70.0%) with poor OHIP-14 scores had taste intensity scores of 3 and 4, respectively, while those with moderate (68.4% and 48.6%) OHIP-14 had scored 1 and 2, respectively.

**Conclusion:**

Olfactory and gustatory disturbances were found to be a long-term feature in post-COVID-19 patients. The blood group is a predisposing factor for persistent smell alterations in post-COVID-19 patients.

## 1. Introduction

The global pandemic of COVID-19 caused by severe acute respiratory syndrome coronavirus-2 (SARS-CoV-2) has shattered millions of lives. Although the exact pathobiology of this viral infection is not fully understood, its typical symptoms include headaches, fever, diarrhoea, cough, shortness of breath, myalgia, sore throat, and rhinorrhea [1, 2]. Additionally, novel severe acute respiratory syndrome coronavirus-2 demonstrates its affinity towards multiple human tissues in neurological, psychiatric, cardiovascular, gastrointestinal, dermatological, and pediatric domains [3–8].

Centers for Disease Control and Prevention (CDC) has added loss of taste or smell to its list of fewer common symptoms that may arise two to 14 days after exposure to the COVID-19 virus. Among the various atypical manifestations of COVID-19 infection, neuro-sensory impairment is often considered an initial warning symptom. Subjective olfactory and gustatory disturbances are also reported before, during, or postrecovery stage of COVID-19. Hence, recognizing the above two chemosensitive dysfunctions in the earlier stages of the disease is crucial for timely identification [9]. Furthermore, numerous studies concluded that anosmia without nasal obstruction and dysgeusia is a pathognomonic sign of COVID-19 infection [10, 11]. Earlier studies have established the link between postviral olfactory symptoms with human rhinovirus, parainfluenza, and Epstein–Barr virus.

A recent meta-analysis of 155 studies across the globe revealed variable prevalence ranging from olfactory (0%–98%) and gustatory dysfunction (0–89%) [12]. Though some patients have reported isolated olfactory or taste disturbance, most complained of combined olfactory and taste alterations. The period between exposure and COVID-19 appearance of symptoms takes 5–6 days with the variation of 1 day to 2 weeks. Most of these patients reported complete recovery; however, evidence suggested prolonged, persistent dysfunction for an extended period, qualifying olfactory and gustatory disturbances as a post-COVID-19 condition. Post-COVID-19 syndrome has been used to refer to any health disorder or disorder that persists for more than a month in people who suffered from mild COVID-19 or were even asymptomatic [13]. There is no substantial unanimity on post-COVID-19 symptoms, and its diagnostic criteria have not been sufficiently established. The varied manifestations of these post-COVID-19 conditions between individuals and the convalescence period it may last are still questionable. The disability associated with the symptoms of the post-COVID-19 syndrome is one of its main characteristics, so the impact it can have on the care and rehabilitation units is considerable. Most studies have focused on analyzing the prevalence of symptoms of a post-COVID-19 syndrome rather than on objective organ involvement. The absence of a standard and accepted definition limits the comparison of findings between the various epidemiological studies [14, 15]. Studies showed that olfactory and gustatory impairments are long-lasting sequelae of COVID-19. Even after 30 to 50 days, patients still suffered from objectifiable smell and taste disorders. The absence of research in this perspective ignited up this piece of an audit [3, 6, 12, 15, 16].

Association of ABO blood group typing with infectious diseases is not just a causal relationship. Various studies often show that blood groups have a direct impact on disease courses and prognosis. There is a well-established correlation between blood groups and numerous viral infections like the Norwalk virus and Hepatitis B [17–19]. A recent meta-analysis of this human respiratory disease with ABO grouping revealed mixed results among various countries [17]. Nonetheless, none of the studies so far assessed the relationship between the severities of olfactory and gustatory disturbances with blood group correlation in COVID-19. Such a relationship could be of significant prognostic value and treatment planning. Furthermore, none of the studies assessed the relationship between olfactory and gustatory disturbance severity with blood group correlation in COVID-19 circumstances, which, if found, could aid in prognosis and treatment planning.

The COVID-19 pandemic has impacted the health-related quality of life of all sectors of the population [20, 21]. To our knowledge, none of the studies explored the long-term effects of olfactory and gustatory impairment in post-COVID-19 patients and its effect on OHRQoL in these subjects. The null hypothesis of this study was that there is no difference in the chemosensitive function in patients with and without post-COVID-19 and there was no association between these dysfunctions and their ABO blood group. Thus, the present study aimed to identify chronic chemosensitive dysfunction in post-COVID-19 patients by objective assessment methods and determine if any association between these dysfunctions and their ABO blood group existed. This study also explored the impact of olfactory and gustatory impairment on OHRQoL among these patients.

## 2. Materials and Methods

This cross-sectional study was conducted among 116 participants who reported to the Outpatient Department of Oral Diagnosis, College of Dentistry, King Khalid University, Saudi Arabia, using convenience sampling from March to September 2021. Ethical approval was obtained from the Institutional Review Board of King Khalid University (IRB/KKUCOD/ETH/2020-21/050). This study adhered to the code of ethics in the Declaration of Helsinki protocol (version 17c, 2004). Complete information about the study was given to the patients in their language, and informed consent was sought before the commencement of the study.

### 2.1. Participants

The inclusion criteria of this study were patients older than 18 years who had COVID-19 infection and became negative in the COVID-19 reverse transcription-quantitative polymerase chain reaction (RT-qPCR) test. These patients were recruited for the study. Patients who had a history of olfactory and taste function alterations before COVID-19 and a history of any systemic disease and smokers were excluded from the study in order to eliminate potential sources of bias.

### 2.2. Variables

The primary investigator (RA) collected demographic data, COVID-19 RT-qPCR test details, and ABO blood group from the patient medical records. Objective assessment of olfaction and gustatory functions and OHRQoL were conducted by the co-investigators of the study (RM, MA, and MH). The olfactory assessment was done using the CCCRC test and odour identification test [22].

### 2.3. Sources of Data and Details of Methods of Assessment

#### 2.3.1. Butanol Threshold Test

In each case, participants were presented with two identical glass bottles simultaneously—one containing water and the other containing a dilute concentration of butanol. They were instructed to occlude one nostril and place the tip of the first bottle immediately beneath the other nostril. The second bottle was then sampled similarly, and the participants had to choose which of the bottles contained something other than water. If the choice was incorrect, the next stronger butanol concentration was presented along with a bottle containing only water. Once the subject correctly identified the same butanol concentration five times in a row, the score was recorded for that nostril. The other nostril was then tested separately, and the scores for both nostrils were averaged to arrive at the final score. The most potent butanol concentration (bottle 0) was 4% butanol in deionized water. Each subsequent dilution (bottles 1–9) was a 1:3 dilution with deionized water. Possible scores ranged from 0 to 9, but all scores seven and higher were scored according to the Connecticut Chemosensory Clinical Research Center (CCCRC) test.

#### 2.3.2. Odour Identification Test

Common household odorants were kept inside opaque jars. Participants chose from a printed list containing the correct items and an equal number of distractor items. Possible scores ranged from 0 to 7 for items that were accurately identified. Scores for both nostrils were averaged to arrive at the final score. Scores for the butanol threshold test and identification were subsequently averaged to arrive at a composite score for olfactory function [22].

#### 2.3.3. Gustatory Function

Gustatory function in the participants was analyzed using a standardized and validated test that investigated the ability to perceive four primary tastes. Gustatory testing covered the four essential gustatory qualities sweet, salty, sour, and bitter. Four solutions for each primary taste were prepared in water using table salt, refined sugar, lemon juice, and unsweetened decaffeinated coffee. According to the correct answers, four categories of taste intensity scores were established: normal (score 4), mild hypogeusia (score 3), moderate hypogeusia (score 2), severe hypogeusia (score 1), and ageusia (score 0) [16]. Subjective loss of smell, loss of taste, and dysgeusia were also recorded for each patient.

#### 2.3.4. Oral Health-Related Quality of Life (OHRQoL)

Oral health-related quality of life was assessed using a standardized OHIP-14 questionnaire. It is a self-reported questionnaire focusing on seven dimensions (functional limitation, pain, psychological discomfort, physical disability, psychological disability, social disability, and handicap) of oral health. Participants were asked to respond on a five-point Likert scale: never coded (score 0), hardly ever (score 1), occasionally (score 2), fairly often (score 3), and very often (score 4). The total score of the OHIP-14 was calculated by adding the scores of the responses for the 14 items. The values of the OHIP-14 score ranged from 0 to 56, with higher scores indicating lower OHRQoL [23].

#### 2.3.5. Statistical Analysis

The collected data were subjected to statistical analysis using appropriate statistical tools. All data were represented as frequency and percentage (for categorical variables). The chi-square test has been used for finding an association between regular exercise and various study variables. If expected frequencies are less than five, either continuity correction for chi-square or Fisher's exact test has been employed. A calculated *p* value less than 0.05 is considered statistically significant. All the analyses were carried out with the help of the commercially available statistical package SPSS v.23 for WINDOWS.

## 3. Results

One hundred sixteen patients were found potentially eligible and examined for the study. One hundred were confirmed eligible and included in the study as they consented to participate and completed the evaluation (twelve participants did not complete the olfactory and gustatory tests, while four did not complete the OHIP-14 questionnaire; hence, their data were not considered for the analysis). The frequency and percentage distribution of patients based on demographic characteristics are presented in Table 1. There were 48% females and 52% males. The majority of the study participants (65%) were below 30 years of age, and more than half (64%) were married. The study participant's educational qualifications included a university degree (61%), high school (28%), secondary school (8%), and primary school (3%). Most of the participants (46%) had O blood group followed by A group (32%), B group (14%), and AB blood group (8%).

Table 2 lists patients' frequency and percentage distribution based on duration after COVID-19, subjective loss of smell and taste, CCCRC score, taste intensity score, dysgeusia, and OHIP-14 score. Most of the study patients (54%) suffered from COVID-19 seven months ago or earlier. About 31% had the disease 4–6 months before the current study, while a small proportion (15%) reported being tested COVID-19 positive within the last three months. About 38% of subjects did not have any olfactory disturbances at the study time, while 8% presented with persistent anosmia at the time of the study. More than half of the patients had either mild, moderate, or severe anosmia when tested. Furthermore, 42% had poor CCCRC scores when screened objectively for olfactory function.

When analyzing the neural impairment in the gustatory domain, 14% reported ageusia. More than half of the study population had mild, moderate, or severe hypogeusia, similar to the olfactory disturbance. Taste intensity score revealed only 20% had a normal taste, while the remaining 80% had any one of the forms of taste alterations and dysgeusia in 12% of the study population. More than half of the study participants (55%) reported poor OHRQoL, and notably, none of the participants had an excellent OHRQoL score.

A statistically significant difference was reported between different ABO blood groups and subjective loss of smell (*p* < 0.05). However, other variables like subjective loss of taste, CCCRC score, and taste intensity score did not show any association with the ABO blood group (Table 3). Subjective loss of taste showed association with the ABO blood group when grouped as positive and negative (Table 4). No statistically significant association was found between most of the evaluated variables (subjective loss of taste, CCCRC score, and dysgeusia) and OHIP-14 score (*p* > 0.05), that is, subjective loss of taste, CCCRC score, and dysgeusia are independent of OHIP-14. However, there was a statistically significant association between taste intensity scores and OHIP-14 scores (*p* < 0.05). Hence, the taste intensity score is dependent on OHIP 14. The majority of the patients (70.8% and 70.0%) with poor OHIP-14 had taste intensity scores of 3 and 4, while those with moderate (68.4% and 48.6%) OHIP-14 had scored 1 and 2 (Table 5).

## 4. Discussion

To the best of our knowledge, this is the first study in Saudi Arabia that reports the presence of chronic chemosensitive dysfunction in post-COVID-19 patients using objective assessment methods and, at the same time, explores the association between these dysfunctions and their ABO blood group and OHRQoL. It was found that most of the participants had some form of olfactory and gustatory impairment following COVID-19 infection and experienced poor OHRQoL. While many aspects of chemosensitive functions were found to be independent of OHRQoL, the taste intensity score was found to be dependent on it.

Various authors have reported the gustatory and olfactory disturbance to have a significant predictive value in the acute phase of COVID-19 [24, 25]. The possible pathophysiology of postinfection chemosensitive disorders could be attributed to either damage to the olfactory/oral epithelium or its central processing pathways [1, 26].

Pathogenic susceptibility of SARS-CoV-2 in olfactory alterations, gustatory domain, and ABO blood groups varies remarkably among different populations [14, 19, 27, 28]. A recent study of 47,910 COVID-19 susceptible individuals showed varied recovery rates [14]. The exact time these symptoms resolve is unknown, necessitating in-depth studies to analyze post-COVID-19 sequelae. Chemosensitive dysfunction, especially olfactory and gustatory, has a more significant effect on domestic incidents and eating disorders, compromising the quality of life significantly [20, 29, 30]. Hence, studies on the epidemiology and post-COVID-19 conditions are the need of the hour to develop evidence-based principles directed to sustain the quality of life among these patients.

In the present study, we had only a handful of studies included in the discussion as very few works evaluated the post-COVID-19 olfactory and gustatory disturbances. Moreover, none of the studies evaluated OHRQoL in post-COVID-19 conditions. In this study, post-COVID-19 conditions of taste and smell disturbances among patients who had recovered from the infection were assessed on an average of 60 days to 210 days after disease onset. Olfactory disorders, either partial or total, were reported in 73% of the population with 8% anosmia. This overall olfactory compromise found in this work did not corroborate with the short-term recovery reported by a multicentric study done in India (85%), France (64%), and Europe (44%), respectively [1, 31, 32].

In terms of hyposmia, the proportion reported in this work is much higher than that reported in similar studies. About 32% of hyposmia conditions are declared in Chinese patients when assessed objectively three months after hospitalization [33]. An Israelian study assessed post-COVID-19 hyposmia among 112 patients between 6 weeks to 6 months after symptoms proclaimed 15% smell change [34]. Further, in a survey among 488 hospitalized COVID-19 positive patients, Chopra and his colleagues found about 13.1% reported the loss of smell 60 days after hospital discharge following the infection [35]. Similarly, another study among the Austrian population found that 19% had anosmia or hyposmia 100 days after symptom onset [36]. A Norwegian study found 12% smell dysfunction with a mean age of 50 years in 41 to 193 days after disease onset [37]. Another prospective geographical cohort study done by Peterson et al. among patients with a mean age of 40 years, 24% reported loss of smell 125 days after disease onset [38].

A recent analysis by Gerkin and his colleagues reported only 40.9% of patients fully recovered after 40 days following the disease onset [39]. Iannuzzi et al. declared that their work could not conclude the full-term recovery of smell two months after rehabilitation [40]. The relatively high percentage of hyposmia may be attributed to the objective nature or validated olfactory tests we used.

Nonetheless, most of the previous literature relied on questionnaires or interviews, which were not quantified in sensory functions. Hence, the proportion reported could not be adequately classified as either partial or complete loss. Awareness of the association between taste alteration and post-COVID-19 is essential for patient management and betterment of public health [13]. Our study found more than half of the subjects had taste alterations, 54% hypogeusia, and 18% ageusia. These findings were similar to the studies done by Peterson et al. [38] (15%) and Sonnweber et al. [36] (19%) but higher than reported by Stavem et al. [37] (10%), Chopra et al. [35] (13%), and Klein et al. [34] (8%).

In the present study, ABO blood grouping was a predisposing factor for persistent anosmia after COVID-19 infection, and a prevalence of an O blood group was seen. However, Mahmud et al. [41] found a higher prevalence of A blood group among COVID-19 patients and no association of ABO blood groups with the clinical presentation or duration of recovery from COVID-19. Similarly, Badedi et al. [42] did not find any significant association between a specific ABO blood group and mortality risk in COVID-19 positive patients. The overall results of this study lead to the rejection of our null hypothesis.

Persistent impairment of olfactory and gustatory chemo senses compromises the quality of life in general and oral health in particular. An association between objective measurement of taste intensity and OHRQoL has been reported in our work. Several research studies that assessed chemosensitive disorder used only subjective measurement, influencing the prevalence rates [43]. In this regard, there are suggestions in the literature for dental treatment during the pandemic periods, especially in a vulnerable population to avoid more serious complications [44].

Although this is a first of its kind study that examines the correlation between post-COVID-19, chemosensitive function, blood group, and oral health-related quality of life, this study has certain limitations. The relatively small sample size of this study is a limitation of this study. Moreover, only patients who reported dental treatment at the outpatient department were included, thus reducing this study's generalizability and external validity. Further long-term multicentric studies are being planned to fully understand the extent of residual impact of these chemosensitive dysfunctions.

In conclusion, this research demonstrates long-term impairment of taste and smell in COVID-19 positive patients identifying it as a definite post-COVID-19 condition in this population. Within the study's limitations, the blood group is a predisposing factor for persistent anosmia in post-COVID-19 patients.

## Figures and Tables

**Table 1:** Frequency and percentage distribution of patients based on demographics.

Variable	Class	Frequency	Percentage
Gender	Female	48	48.0
	Male	52	52.0
	Total	100	100.0

Age	Less than 30	65	65.0
	30–40	18	18.0
	Greater than 40	17	17.0
	Total	100	100.0

Marital status	Married	64	64.0
	Unmarried	36	36.0
	Total	100	100.0

Education	Primary school	3	3.0
	Secondary school	8	8.0
	High school	28	28.0
	College/university	61	61.0
	Total	100	100

ABO blood group	A	32	32.0
	AB	8	8.0
	B	14	14.0
	O	46	46.0
	Total	100	100.0

**Table 2:** Frequency and percentage distribution of patients based on duration after COVID-19, subjective loss of smell and taste, CCCRC score, taste intensity score, dysgeusia, OHIP-14 score.

Variable	Class	Frequency	Percentage
Duration after recovering from COVID-19	Less than or equal to 3 months	15	15.0
	4–6 months	31	31.0
	Greater than or equal to 7 months	54	54.0
	Total	100	100.0

Subjective loss of smell	Anosmia	8	8.0
	Severe hyposmia	14	14.0
	Moderate hyposmia	28	28.0
	Mild hyposmia	12	12.0
	Normal	38	38.0
	Total	100	100.0

Subjective loss of taste	Ageusia	14	14.0
	Severe hypogeusia	15	15.0
	Moderate hypogeusia	27	27.0
	Mild hypogeusia	12	12.0
	Normal	32	32.0
	Total	100	100.0

CCCRC score	Good	58	58.0
	Poor	42	42.0
	Total	100	100.0

Taste intensity score	1	19	19.0
	2	37	37.0
	3	24	24.0
	4	20	20.0
	Total	100	100.0

Presence of dysgeusia	No	88	88.0
	Yes	12	12.0
	Total	100	100.0

OHIP-14 score	Poor	56	56.0
	Moderate	44	44.0
	Total	100	100.0

**Table 3:** Chi-square test of association between ABO blood groups and chronic chemosensitive dysfunction in post- COVID-19 patients (fishers exact test).

Chemosensitive dysfunction	ABO blood groups				Chi-square	df	*p* value
	A	AB	B	O			
*Subjective loss of smell*
Anosmia	2 (25.0%)	1 (12.5%)	2 (25.0%)	3 (37.5%)	24.362	12	0.006^*∗∗*^
Severe hyposmia	3 (21.4%)	1 (7.1%)	2 (14.3%)	8 (57.1%)			
Moderate hyposmia	13 (46.4%)	4 (14.3%)	3 (10.7%)	8 (28.6%)			
Mild hyposmia	9 (75.0%)	0 (0.0%)	1 (8.3%)	2 (16.7%)			
Normal	5 (13.2%)	2 (5.3%)	6 (15.8%)	25 (65.8%)			
*Subjective loss of taste*					13.174	12	0.300NS
Ageusia	3 (21.4%)	1 (7.1%)	3 (21.4%)	7 (50.0%)			
Severe hypogeusia	5 (33.3%)	2 (13.3%)	2 (13.3%)	6 (40.0%)			
Moderate hypogeusia	12 (44.4%)	3 (11.1%)	3 (11.1%)	9 (33.3%)			
Mild hypogeusia	7 (58.3%)	0 (0.0%)	1 (8.3%)	4 (33.3%)			
Normal	5 (15.6%)	2 (6.2%)	5 (15.6%)	20 (62.5%)			
*CCCRC score*					1.806	3	0.619 NS
Good	18 (31.0%)	3 (5.2%)	9(15.5%)	28(48.3%)			
Poor	14 (33.3%)	5 (11.9%)	5 (11.9%)	18 (42.9%)			
*Dysgeusia*					0.952	3	0.839 ^NS^
No	29 (33.0%)	7 (8.0%)	13 (14.8%)	39 (44.3%)			
Yes	3 (25.0%)	1 (8.3%)	1 (8.3%)	7 (58.3%)			
*Taste intensity score*					13.888	9	0.096 NS
1	6 (31.6%)	1 (5.3%)	3 (15.8%)	9 (47.4%)			
2	6 (16.2%)	3 (8.1%)	4 (10.8%)	24 (64.9%)			
3	9 (37.5%)	2 (8.3%)	4 (16.7%)	9 (37.5%)			
4	11 (55.0%)	2 (10.0%)	3 (15.0%)	4 (20.0%)			

NS: not significant (*p* > 0.05) ^*∗*^: significant (*p* < 0.05).

**Table 4:** Chi-square test of association between positive and negative blood groups and chronic chemosensitive dysfunction in post-COVID-19 patients (fishers exact test).

Chemosensitive dysfunction	ABO blood groups		Chi-square	df	*p* value
	Positive	Negative			
*Subjective loss of smell*			6.058	4	0.127 ^NS^
Anosmia	8 (100.0%)	0 (0.0%)			
Severe hyposmia	12 (85.7%)	2 (14.3%)			
Moderate hyposmia	28 (100.0%)	0 (0.0%)			
Mild hyposmia	10 (83.3%)	2 (16.7%)			
Normal	32 (86.5%)	5 (13.5%)			
*Subjective loss of taste*			7.835	4	0.049^*∗*^
Ageusia	11 (84.6%)	2 (15.4%)			
Severe hypogeusia	13 (86.7%)	2 (13.3%)			
Moderate hypogeusia	27 (100.0%)	0 (0.0%)			
Mild hypogeusia	9 (75.0%)	3 (25.0%)			
Normal	30 (93.8%)	2 (6.2%)			
*CCCRC score*			0.233	1	0.630 ^NS^
Good	53 (93.0%)	4 (7.0%)			
Poor	37 (88.1%)	5 (11.9%)			
*Taste intensity score*			3.226	3	0.385 ^NS^
1	18 (94.7%)	1 (5.3%)			
2	34 (94.4%)	2 (5.6%)			
3	22 (91.7%)	2 (8.3%)			
4	16 (80.0%)	4 (20.0%)			
*Dysgeusia*			0.000	1	1.000 ^NS^
No	79 (90.8%)	8 (9.2%)			
Yes	11 (91.7%)	1 (8.3%)			

NS: not significant (*p* > 0.05) ^*∗*^: significant (*p* < 0.05).

**Table 5:** Chi-square test of association between OHIP14 (oral health index profile) and chronic chemosensitive dysfunction in post-COVID-19 patients (fishers exact test).

Chemosensitive dysfunction	OHIP14		Chi-square	df	*p* value
	Poor	Moderate			
*Subjective loss of smell*
Anosmia	5 (62.5%)	3 (37.5%)	4.168	4	0.393^NS^
Severe hyposmia	6 (42.9%)	8 (57.1%)			
Moderate hyposmia	13 (46.4%)	15 (53.6%)			
Mild hyposmia	9 (75.0%)	3 (25.0%)			
Normal	23 (60.5%)	15 (39.5%)			
Subjective loss of taste			6.624	4	0.157 NS
Ageusia	9 (64.3%)	6 (35.7%)			
Severe hypogeusia	8 (53.3%)	7 (46.7%)			
Moderate hypogeusia	11 (40.7%)	16 (59.3%)			
Mild hypogeusia	10 (83.3%)	2 (16.7%)			
Normal	18 (56.2%)	14 (43.8%)			
CCCRC score			0.038	1	0.845 NS
Good	32 (55.2%)	26 (44.8%)			
Poor	24 (57.1%)	18 (42.9%)			
Taste intensity score			8.657	3	0.034^*∗*^
1	6 (31.6%)	13 (68.4%)			
2	19 (51.4%)	18 (48.6%)			
3	17 (70.8%)	7 (29.2%)			
4	14 (70.0%)	6 (30.0%)			
Dysgeusia			0.630	1	0.427 ^NS^
No	48 (54.5%)	40 (45.5%)			
Yes	8 (66.7%)	4 (33.3%)			

NS: not significant (*p* > 0.05) ^*∗*^: significant (*p* < 0.05).

## Data Availability

Data will be made available on request to the corresponding author.
